# Separating content-specific retrieval from post-retrieval processing

**DOI:** 10.1016/j.cortex.2016.10.003

**Published:** 2017-01

**Authors:** Amie N. Doidge, Lisa H. Evans, Jane E. Herron, Edward L. Wilding

**Affiliations:** a College of Life and Environmental Sciences, School of Psychology, Exeter University, UK; b Cardiff University Brain Research Imaging Centre (CUBRIC), School of Psychology, Cardiff University, UK; c School of Psychology, Nottingham University, UK

**Keywords:** Context reinstatement, Episodic memory, Content-specific retrieval, Recollection

## Abstract

According to cortical reinstatement accounts, neural processes engaged at the time of encoding are re-engaged at the time of memory retrieval. The temporal precision of event-related potentials (ERPs) has been exploited to assess this possibility, and in this study ERPs were acquired while people made memory judgments to visually presented words encoded in two different ways. There were reliable differences between the scalp distributions of the signatures of successful retrieval of different contents from 300 to 1100 ms after stimulus presentation. Moreover, the scalp distributions of these content-sensitive effects changed during this period. These findings are, to our knowledge, the first demonstration in one study that ERPs reflect content-specific processing in two separable ways: first, via reinstatement, and second, via downstream processes that operate on recovered information in the service of memory judgments.

## 1 Introduction

There is strong support for the claim that the neural systems engaged during retrieval of contextual information vary according to the content that is retrieved (Johnson and Rugg, 2007, Kahn et al., 2004, Wheeler and Buckner, 2003, Wheeler et al., 2000, Wheeler et al., 1995, Woodruff et al., 2005). This evidence-base has frequently been considered in terms of the reinstatement of neural activity that was engaged at the time information was encoded (McClelland et al., 1995, Mesulam, 1998, Norman and O'Reilly, 2003). By these accounts, episodic retrieval involves the recapitulation of patterns of cortical activity that occurred at encoding, with representations in the hippocampus being important for successful reinstatement to occur (Norman & O'Reilly, 2003).

Much of the empirical support for content-dependence at the time of retrieval has come from functional magnetic resonance imaging (fMRI) studies (Johnson & Rugg, 2007). The limited temporal resolution of this imaging technique, however, means that it is not possible to distinguish between activity that is a direct reflection of recovery of different kinds of content, and activity reflecting processes operating over recovered contents (Johnson and Rugg, 2007, Yick and Wilding, 2008). Event-related potentials (ERPs) are well-placed to provide data relevant to this proposed process separation because they index neural activity in real time.

The sensitivity of ERPs to recovery of different contents has, however, been determined only partially. What is uncontroversial is the claim that ERPs index several distinct memory processes (Friedman and Johnson, 2000, Wilding and Ranganath, 2012). The most common approach to identifying these has been to analyze ERP old/new effects, which are differences between the neural activities elicited by old (studied) and new (unstudied) stimuli attracting accurate memory judgments (Rugg, 1994). Several old/new effects have been identified on the basis of differences between their time courses, scalp distributions and sensitivities to experimental manipulations. The last of these factors has enabled claims to be made about the functional significance of particular effects (Wilding & Sharpe, 2003).

One of the most frequently reported ERP old/new effects is the left-parietal effect. This consists of a greater relative positivity for old in comparison to new items and is largest over left-posterior scalp locations between 500 and 800 milliseconds (msec) after stimulus onset (Allan, Wilding, & Rugg, 1998). The functional significance of this effect has been assessed in many studies (Wilding & Ranganath, 2012). The consensus is that it acts as an index of the process of recollection – recovery of qualitative information from a prior episode – and that it does so in a graded fashion (Vilberg & Rugg, 2009a).

Perhaps the first compelling evidence for this functional claim was provided by Smith (1993), who demonstrated that this old/new effect was larger when people made ‘Remember’ rather than ‘Know’ judgments to studied words. These data support the link between the parietal ERP old/new effect and recollection because it has been demonstrated that recollection is associated to a greater degree with Remember than with Know responses (Yonelinas, 2002, Yonelinas and Jacoby, 2012).

These findings in studies where subjective reports were offered (for a variant, see Vilberg, Moosavi, & Rugg, 2006), have been complemented by studies where forced choice context judgments have been required. Wilding, Doyle, and Rugg (1995) and Wilding and Rugg (1996) demonstrated that the left parietal ERP old/new effect was larger when people made accurate judgments about the context in which words had been encountered in a prior study phase than when they were able to identify words as having been studied but made inaccurate context judgments. These data points are part of the basis for the claim that the left-parietal ERP old/new effect indexes the quality or volume of contextual information that is recovered. Additional support for this claim comes from findings that the effect in question is larger when two rather than one contextual elements are recovered (Vilberg and Rugg, 2008, Vilberg and Rugg, 2009a, Vilberg and Rugg, 2009b, Wilding, 2000).

Further evidence consistent with the link between this effect and recollection comes from pharmacological and patient studies. Midazolam-induced and lesion-induced impairments in recollection are associated with attenuated left-parietal ERP old/new effects (Curran, DeBuse, Woroch, & Hirshman, 2006; Duzel et al., 2001, Vargha-Khadem et al., 1997). Finally, the period over which the left-parietal ERP old/new effect is prominent is noteworthy. In behavioral assessments of the time course of the contribution of recollection to memory judgments, discrimination levels comparable to those observed in ERP studies are linked with reaction times exceeding 800 ms post-stimulus (Gronlund et al., 1997, Hintzman and Caulton, 1997, Yonelinas and Jacoby, 1994).

These data points, acquired under a range of different circumstances and from different populations, argue strongly for the coupling between this effect and the process of recollection (Mecklinger, 2000, Rugg and Curran, 2007, Wilding and Ranganath, 2012). Moreover, and critically for present purposes, in the studies described above, and in others where ERPs have been acquired from young adults during tasks that can be supported by recollection, different kinds of contextual information have been recovered and the timing and left-parietal maximum of the effect has remained largely unchanged (Cruse & Wilding, 2009; Cycowicz et al., 2001, Duarte et al., 2004, Johansson et al., 2004, Nessler and Mecklinger, 2003, Trott et al., 1997, Wilding et al., 2005). This outcome is consistent with the view that the left-parietal old/new effect is not content-sensitive (Donaldson and Curran, 2007, Johnson et al., 2008, MacKenzie and Donaldson, 2007).

Other ERP old/new effects have, however, indicated a degree of sensitivity to the contents of what is retrieved, with the strongest examples stemming from contrasts between the old/new effects elicited by unfamiliar faces and other kinds of content. Yick and Wilding (2008) compared the ERP old/new effects elicited by faces and words in a task requiring old/new recognition memory judgments. A left-parietal ERP old/new effect was evident for faces as well as for words. For faces only, an old/new effect in the same epoch extended anteriorly to central and frontal scalp locations. Because this frontal distribution was specific to faces and appeared to onset at least as early as the left-parietal ERP old/new effect, the authors observed that their findings were consistent with the view that it reflected the on-line recovery of content associated with faces but not with words (which does not of course necessitate that the effect is specific to faces: Galli and Otten, 2010, MacKenzie and Donaldson, 2007, MacKenzie and Donaldson, 2009, Schloerscheidt and Rugg, 1997).

These outcomes attest to the content-sensitivity of ERP old/new effects, in so far as they diverge when words or faces are stimuli. The evidence, however, for broader sensitivity of ERPs to other kinds of content – hence their potential to contribute to general accounts of context reinstatement – is limited. In one notable study, Johnson et al. (2008) showed participants studied and unstudied words. In a prior encoding phase each studied word had been encountered in one of two tasks. One task was to incorporate individually presented words into a sentence. The other was to imagine how an object denoted by a word might be located appropriately in a background image. As in the work with faces, divergences between the old/new effects associated with recovery of which task had been completed at study occurred in the 500–800 msec post-stimulus epoch. The old/new effects associated with recovery of words that were incorporated into sentences extended anteriorly to a greater extent than was the case for words that might be incorporated into images. Importantly, however, participants identified words as having been studied in the sentence encoding task markedly more often than was the case for words studied in the picture location task. In the absence of matched performance it remains a possibility that the scalp distribution differences between effects are linked to the relative difficulty of the retrieval tasks, and perhaps differences between the time courses of neural activities due to differences in memory strengths, rather than to recovery of different contents.

The experiment described here was designed to assess the sensitivity of ERPs to content-specific retrieval under circumstances where response accuracy was matched, and in which faces were not included as stimuli. Participants initially saw words (all concrete nouns). Each word was encountered in one of two encoding conditions. In one, the word was followed by a picture of the object denoted by the word. In the other, the word was followed by an empty frame, which cued participants to imagine an image of the object denoted by the word. In subsequent test phases, participants saw studied and unstudied words one at a time (Johnson & Raye, 1981). The task response requirements involved separating studied words from unstudied words, and separating studied words according to the encoding condition. At issue in this experiment is whether, and if so when, ERP old/new effects differ as a function of whether participants accurately retrieve items they perceived or imagined at encoding, hence the extent to which ERPs can provide insights into neural reinstatement accounts for different combinations of stimuli and encoding tasks.

## 2 Methods

### 2.1 Participants

These were 54 individuals (10 male) recruited from Cardiff University. They were each paid £10/h. All provided written informed consent, spoke English as a first language, had normal or corrected-to-normal vision, were right-handed, had no prior diagnosis of dyslexia and reported that they were not taking psychotropic medication. Six participants were excluded from analyses for an estimate of discrimination below .1 (5 participants, see text below for the discrimination measure that was employed) or insufficient ERP trials contributing to an average (<16 artefact free trials in at least one category of interest: 1 participant). Of the remaining 48 participants (mean age = 20.50 years; range 18–27 years), 8 were male. Ethical approval for the study was obtained from the Cardiff University School of Psychology Ethics Committee.

### 2.2 Stimuli

Four hundred and eighty six picture-word pairs were selected from the International Picture Naming Project database (http://crl.ucsd.edu/experiments/ipnp/). The words had a frequency range of 1–9 per million, each had 3–10 letters, and all were presented in white on a black background in Times New Roman font at a viewing distance of 1.2 m. Words subtended maximum visual angles of 0.8° (vertical) and 5.6° (horizontal). The pictures were black line drawings on a white background. They subtended maximum visual angles of 12.3° (vertical) and 10.5° (horizontal). The mean percentage naming frequency, according to the values reported in the database, was 86%.

### 2.3 Design

Three hundred and sixty picture-word pairs were selected randomly from the larger set. These were sorted randomly into three lists (120 picture-word pairs per list). Study lists were formed from two of these lists. Test lists comprised all three lists. The remaining 126 picture-word pairs from the larger set were used as filler items; 26 were shown at the beginning and 100 at the end of each study list. The filler words were constant for each participant. Three different combined study-test lists were constructed so that across the combined lists words appeared at test as new (unstudied) words and as studied words that had been encountered in either study condition (*perceive* and *imagine,* respectively; see below). Test lists were divided, with 180 items in each half, and the two halves contained an equal number of new words and words shown at study in each condition. There was a 1 h filled delay between the study and test phases, during which participants completed a number of psychometric measures. The values obtained for these measures are not reported here. The EEG cap was also applied in this period.

**Fig. 1:**
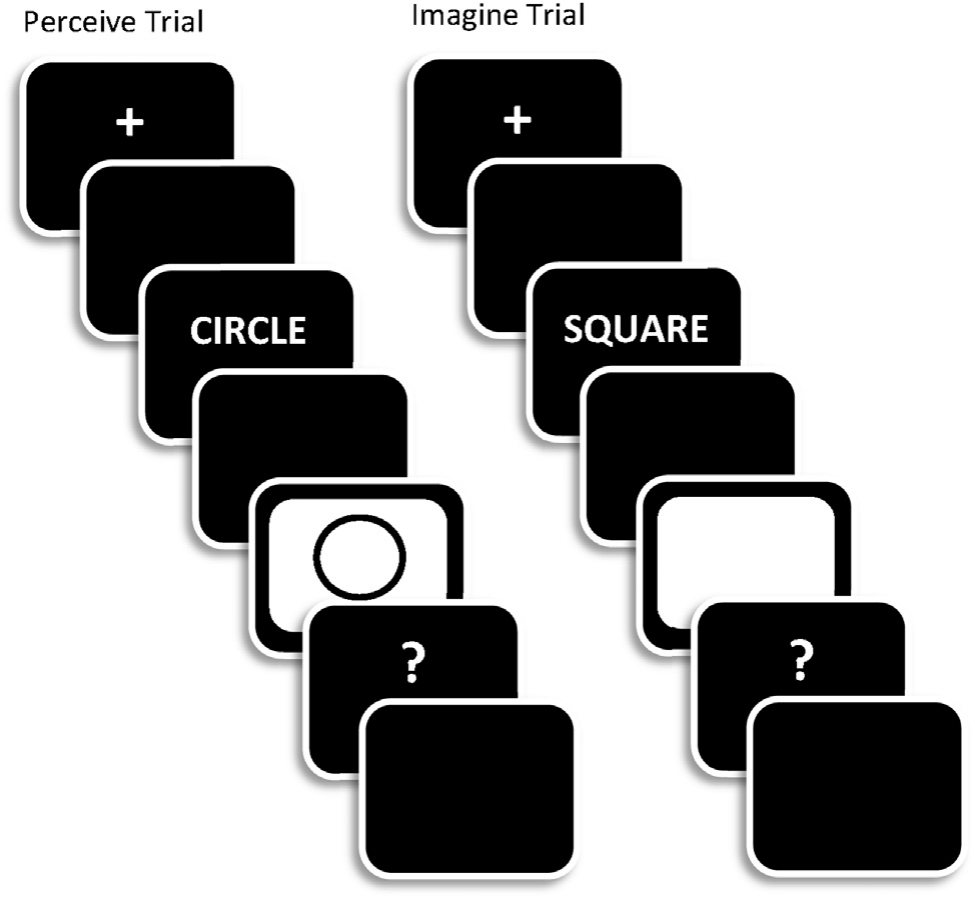
A schematic representation of the trial sequences for Perceive (left-hand side) and Imagine (right-hand side) trials in the study phases of the experiment. Trial timings are described in the text.

All study trials started with a fixation cross (500 msec), a blank screen (300 msec), then a word (300 msec) followed by a blank screen (150 msec) and finally a white frame (1500 msec; see Fig. 1). In the study phase there were two trial-types. On *perceive* trials, a black and white line drawing of the object denoted by each word was presented within (and simultaneously with) the white frame. On *imagine* trials, only the white frame was presented and participants were asked to imagine a line drawing of the object denoted by the preceding word. The white frame and picture were replaced by a question mark. When this appeared, participants indicated the quality of the perceived or imagined image via key-press (response options: good, fair, poor). These responses were made with the index, middle and ring fingers of the right hand. The trial was terminated by a participant key press or when 3000 msec from the onset of the question mark had elapsed. Under both sets of circumstances the screen was then blanked for 1000 msec before the onset of the next trial.

**Fig. 2:**
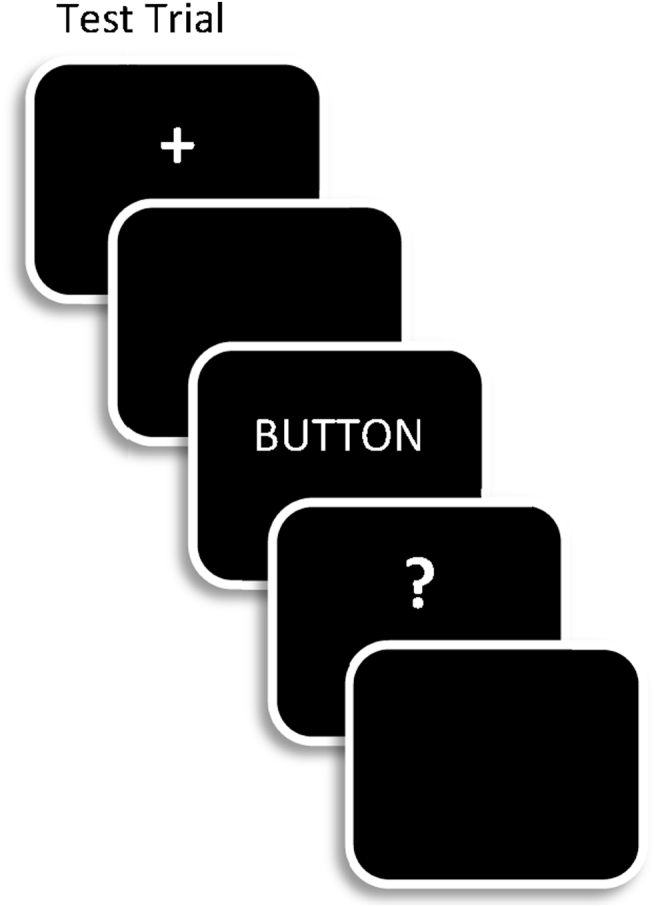
A schematic representation of the trial sequence in the test phases of the experiment. Trial timings are described in the text.

All test trials started with a fixation cross (500 msec), followed by a blank screen (300 msec), and then a word (300 msec duration; see Fig. 2). The word was replaced by a question mark, during which time participants were asked to make one of two key presses; respond on one key for words encountered in one of the two encoding conditions (designated as targets) and on the other key to new (unstudied) words as well as to words from the alternate encoding condition (designated as non-targets). The target designation was changed at the end of the first half of the test phase. The screen was blanked immediately following the participant response or after 3000 msec if a response was not made by that point. The screen then remained blank for 1000 msec before the next trial started. Responses were made with the index finger of each hand. The order in which study and test items within each list were presented was determined randomly for each participant, and the order of target designation was counterbalanced, as were the hands used for responses at study and test.

### 2.4 Electroencephalogram (EEG) acquisition

EEG data were recorded from 25 silver/silver chloride electrodes embedded in an elasticated cap and from two further electrodes placed on the left and right mastoid processes. Recording sites were based on the International 10–20 system (Jasper, 1958) and comprised midline (Fz, Cz, Pz), fronto-polar (Fp1/Fp2), frontal (F7/8, F5/6, F3/4), central (T7/8, C5/6, C3/4), parietal (P7/8, P5/6, P3/4) and occipital sites (O1/2). Vertical and horizontal eye movements were recorded from additional bipolar electrodes placed above and below the right eye (vertical electro-oculargram [VEOG]) and on the outer canthi (horizontal electro-oculargram [HEOG]). EEG was recorded at 250 Hz (4 msec/point) relative to an average reference. Data were re-referenced offline to the average signal at the two mastoids. EEG and EOG were recorded with a bandwidth of .03–40 Hz. Trials containing large EOG or other artefacts were rejected, as were trials containing A/D saturation or baseline drift exceeding ±75 μV. EOG activities reflecting eye-blinks were corrected using the algorithm introduced by Gratton, Coles, and Donchin (1983). Epochs were 1700 msec in length, including a 200 msec pre-stimulus baseline, relative to which all mean amplitude measures were taken.

## 3 Results

All ANOVAs reported below are corrected for non-sphericity using the Greenhouse–Geisser correction when appropriate (Greenhouse and Geisser, 1959, Winer, 1971). Corrected degrees of freedom as well as the accompanying epsilon values (*ε*) are shown in the text. Only effects involving the factor of response category (spanning correct responses to targets, non-targets and new test words) are reported, and significant main effects and interactions are not reported when they are moderated by higher order interaction terms.

### 3.1 Behavior

The probabilities of correct responses to targets, non-targets and new words are shown in Table 1, split according to target designation (imagine, perceive) and accompanied by the associated reaction times (RTs). For both target designations the likelihood of a target response to a target [*p*(target|target)] was reliably greater than a target response to a non-target [*p*(target|non-target)] and a new word [*p*(target|new): smallest *t*(47) = 23.77,*p* < .001]. A 2×2 repeated measures ANOVA with factors of target designation (imagine and perceive) and discrimination measure [*p*(target|target) – *p*(target|non-target) and *p*(target|target) – *p*(target|new)] revealed only that discrimination between targets and new test words was superior to discrimination between targets and non-targets [*F*(1,47) = 49.26,*p* < .001].

A 3×2 repeated measures ANOVA of RTs for correct responses for the three response categories separated by target designation revealed an interaction between category and designation [*F*(1.8,84.4) = 8.51,*p* < .01,*ε* = .90]. Pairwise Bonferroni-corrected *t*-tests (adjusted *α* = .006) for each type of correct response across the two target designations revealed only that RTs for targets under the perceive target designation were faster than RTs for targets under the imagine designation [*t*(47) = 3.83,*p* < .001].

### 3.2 ERP results

The initial analyses of the ERP data were for the 500–800 msec epoch. This is the epoch in which ERP old/new effects have most often been shown to vary with content (Galli and Otten, 2010, MacKenzie and Donaldson, 2007, MacKenzie and Donaldson, 2009, Yick and Wilding, 2008, Yick and Wilding, 2014). In separate initial ANOVAs for each target designation, the mean amplitudes associated with correct judgments to targets^1^ were contrasted with those associated with correct rejections. In both contrasts, and in all of the analyses reported below, the factor of site was included (25 levels; FP1/2, F7/8, F5/6, F3/4, Fz, T7/8, C5/6, C3/4, Cz, P7/8, P5/6, P3/4, Pz, O1/2) and in both a reliable interaction between response category and site was obtained [*F*(4.3,201.7) = 7.04,*p* < .001,*ε* = .18; *F*(4.2,197.4) = 8.41,*p* < .001,*ε* = .18 for imagine and perceive items, respectively].

**Fig. 3:**
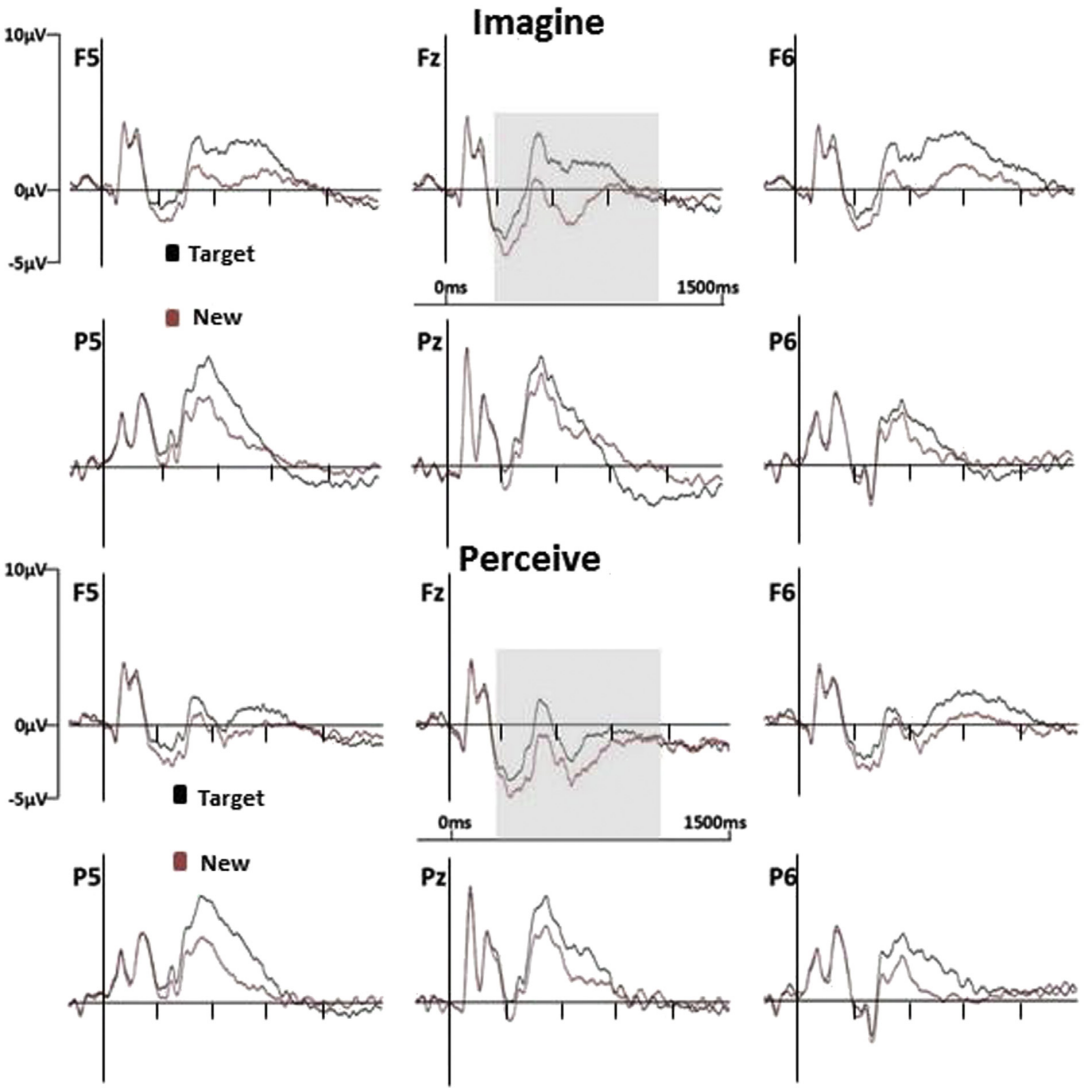
Grand average ERPs elicited by correct responses to Targets and to New test words in the Imagine (upper portion) and Perceive (lower portion) target designations. Data are shown for six representative electrode locations at midline and left and right frontal (Fz, F5, F6) and posterior (Pz, P5, P6) scalp sites. The grey translucent inserts indicate the time periods in which reliable ERP old/new effects were evident (from 300 to 1100 msec in both designations). Waveforms are low-pass filtered at 30 Hz for purposes of presentation.

**Fig. 4:**
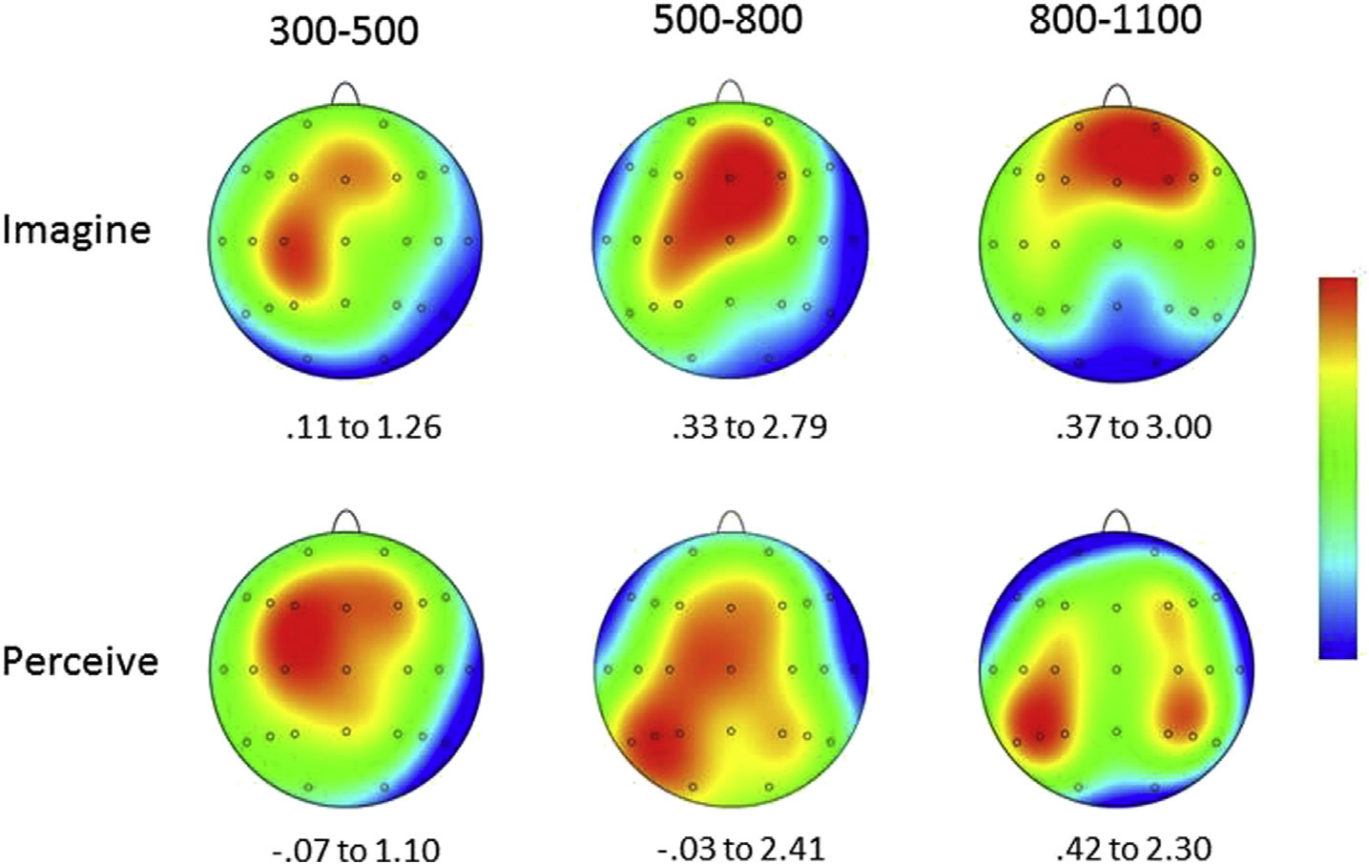
Topographic maps showing the scalp distributions of the Target ERP old/new effects in the Imagine (upper portion) and Perceive (lower portion) conditions for three post-stimulus epochs: 300–500, 500–800 and 800–1100 msec. The maps are the results of spherical spline interpolations over the difference scores obtained by subtracting mean amplitudes associated with correct responses to new test words from those associated with correct responses to targets. Maximum and minimum voltages (μV) are displayed below each map and can be interpreted via the centrally located colour bar.

The purpose of these analyses was to determine that reliable old/new effects were evident in both conditions and that in at least one case there was an interaction between category and scalp location. These criteria were pre-requisites for further analyses in which the sensitivity of ERP old/new effects to the contents of retrieval was investigated by contrasting the difference scores obtained by subtracting mean amplitudes associated with correct rejections from those associated with correct target judgments. Site was again included as a factor (levels as indicated above) along with target designation, and a reliable interaction was revealed [*F*(6.2,291.8) = 5.68,*p* < .001,*ε* = .26]. Moreover, this interaction remained reliable when the analysis was conducted over data rescaled using the min–max method [*F*(6.0, 282.1) = 5.90,*p* < .001,*ε* = .25].^2^ The reason for this outcome is the markedly more anterior distribution of the old/new effects in the imagine than the perceive target designation which can be seen clearly in waveforms in Fig. 3 and the spherical spline interpolations in Fig. 4. Following these outcomes, two additional sets of analyses were conducted for the data from the 300–500 and 800–1100 msec post-stimulus epochs. ERP old/new effects have consistently been reported in these epochs in previous work (Wilding and Rugg, 1996, Wilding and Rugg, 1997), and the analysis strategy for the effects in these epochs matched that used for the 500–800 msec epoch.

#### 3.2.1 300–500 msec

The analysis of the old/new effects for the imagine target designation revealed a significant interaction between response category and site [*F*(4.2,196.2) = 2.63,*p* = .034,*ε* = .17]. For the perceive target designation a main effect of response category was obtained [*F*(1,47) = 10.19,*p* = .003]. When the old/new difference scores were contrasted across designations the interaction between designation and site was reliable [*F*(6.3,296.2) = 2.77,*p* = .011,*ε* = .26], suggesting that the old/new effects differ qualitatively in this epoch. This possibility was confirmed as the interaction term remained significant after rescaling [*F*(6.0,283.3) = 2.86,*p* = .01,*ε* = .25]. These outcomes reflect the fact that while both old/new effects are distributed over mid-frontal and centro-parietal scalp, and with a degree of left-lateralisation, the distribution of the imagine old/new effect is somewhat more focal than that of the perceive effect, as can be seen in Fig. 3, Fig. 4.

#### 3.2.2 800–1100 msec

Significant interactions between response category and site were also obtained for this epoch when the old/new effects were first analyzed separately for the imagine [*F*(5.5,256.4) = 8.68,*p* < .001,*ε* = .23] and perceive target designations [*F*(5.4,252.7) = 3.14,*p* = .007,*ε* = .22]. When the difference scores were analyzed across designations a significant interaction between site and target designation was obtained for the data before [*F*(7.1,334.8) = 12.12,*p* < .001,*ε* = .30] and after rescaling [*F*(7.0,327.1) = 11.53,*p* < .001,*ε* = .29]. The significant results reflect the fact that the anterior maximum of the imagine old/new effect contrasts markedly with the posterior-parietal maxima of the perceive effect (see Fig. 3, Fig. 4).

#### 3.2.3 Analyses across epochs

In light of the evidence for qualitative differences between the old/new effects in the perceive and imagine target designations, two further analyses on rescaled data were conducted. These were designed to assess whether the qualitative differences changed across epochs. They consisted of paired contrasts between pairs of epochs: 300–500 versus 500–800 msec, 500–800 versus 800–1100 msec and 300–500 versus 800–1100 msec.

The divergences between the scalp distributions were not reliably different for the 300–500 and 500–800 msec epochs. An interaction term involving the factors of epoch and designation was reliable for the 500–800 versus 800–1100 msec contrast: target designation × epoch × site [*F*(5.9,276.0) = 9.31,*p* < .0001,*ε* = .25]. The topographic maps in Fig. 4 show that the imagine old/new effect is largest at frontal locations in both the 500–800 and 800–1100 msec epochs. The distribution extends markedly over left-central and left-posterior scalp only in the earlier epoch. In the perceive target designation, by contrast, the largest differences remain at posterior locations from 500 to 1100 msec, with a more posterior distribution overall from 800 msec onwards. For the 300–500 versus 800–1100 msec contrast, the reliable epoch × site interaction [*F*(6.8,318.3) = 2.59, *p* < .025] indicated that the old/new effects were qualitatively different in the two epochs. The higher-order interaction with target designation approached significance: target designation × epoch × site [*F*(6.0,279.9) = 2.00,*p* = .066,*ε* = .25].

#### 3.2.4 Analyses of ERPs elicited by new (unstudied) test items

In a final analysis, the ERPs elicited by correct rejections were subjected to paired contrasts within each epoch. The analyses included the factors of target designation (imagine/perceive) and site (25 levels; FP1/2, F7/8, F5/6, F3/4, Fz, T7/8, C5/6, C3/4, Cz, P7/8, P5/6, P3/4, Pz, O1/2). The only significant outcomes were main effects of target designation from 500 msec onwards: 500–800 msec *F*(1,47) = 8.78,*p* < .01; 800–1100 msec *F*(1,47) = 12.02,*p* < .01. These reflect a small and sustained greater relative positivity for the ERPs elicited by correct rejections in the imagine target designation relative to the perceive designation.^3^

## 4 Discussion

The main aim of this investigation was to assess the sensitivity of event-related potentials (ERPs) to the contents of episodic retrieval, and the time-periods in which any such sensitivities were evident. By doing this, it was possible to assess evidence for context reinstatement. Strong demonstrations of the sensitivity of ERPs to recovery of different kinds of content have to date been restricted to contrasts between words, faces and objects (Galli and Otten, 2010, MacKenzie and Donaldson, 2007, MacKenzie and Donaldson, 2009, Yick and Wilding, 2008, Yick and Wilding, 2014) and tying these effects confidently to recollection has not always been straightforward because some tasks did not include a manipulation that explicitly required judgments about study context (Galli and Otten, 2010, Yick and Wilding, 2008).

In this experiment words were first studied in encoding conditions in which participants either saw pictures (the perceive condition) or were asked to imagine pictures when cued by a visually presented word (the imagine condition). In two subsequent test phases participants responded on one key to words shown in one of the two study contexts (targets) and on another key to new words, as well as to words from the alternate study context (non-targets). Target designation (perceive/imagine) changed across test phases. The accuracy of target judgments was high (∼75%), suggesting that a substantial proportion of correct responses to targets were based on recollection of information from the study episode.

Reliable old/new effects were evident in both target designations between 300 and 1100 msec post-stimulus. The analyses of the scalp distributions of these effects revealed qualitatively different topographic distributions in the 300–500, 500–800 and 800–1100 msec post-stimulus epochs. These outcomes are consistent with the view that not entirely the same neural – hence cognitive-processes were engaged in the separate target designations in each of these time periods. Moreover, the analyses of the ERPs elicited by correct rejections and separated by target designation revealed only a greater relative positivity associated with the imagine designation from 500 msec onwards. Analyses restricted to neural activity associated with correct rejections have been identified as a means of isolating processes linked to retrieval attempts (Bridger et al., 2009, Rosburg et al., 2011, Wilding and Nobre, 1999, Wilding and Nobre, 2001). The assumption is that these contrasts are not contaminated with differences in study history, as is the case for neural activity elicited by old test items (Rugg and Wilding, 2000, Wilding, 1999). Consequently, any differences that emerge from these contrasts are candidates for processes linked to attempts to recover and make decisions about task-relevant information. For present purposes, the key outcome in this experiment is the absence of interactions involving site in the contrast between correct rejections when they are separated according to target designation. This evidence for quantitative differences only provides a reassurance that the qualitative differences between ERP old/new effects that have been reported here can be linked to the recovery of different contents and hence license considerations relevant to cortical reinstatement.

There was also evidence that the distributions of the differences between the old/new effects for the two target designations changed with time. The distributions of the differences were reliably different between the 500–800 and 800–1100 msec epochs and approached significance for the contrast between the 300–500 and 800–1100 msec epochs. Broadly, and as Fig. 3, Fig. 4 illustrate, these differences reflect a consistently more anterior distribution for old/new effects in the imagine relative to the perceive condition from 500 msec onwards and a somewhat more focal distribution in the 300–500 msec epoch.

What are the implications of these findings for context reinstatement accounts? In their consideration of the time-course of content-specific indices of retrieval processing, Johnson et al. (2008) noted that evidence for content-specific retrieval processing might reflect recovery of different episodic content or processes that operate on that content, and that data acquired in fMRI studies could not distinguish between these possibilities because of the temporal characteristics of the haemodynamic response. They also argued that a separation between these two classes of processes could be achieved with ERPs, using the left-parietal ERP old/new effect as a key temporal reference point. Under the assumption that this effect acts as a generic index of recollection (see Introduction), they argued that processes preceding this effect were candidates for content-specific retrieval, while processes succeeding or acting in parallel with the effect could be attributed more readily to post-retrieval processing operations (Yick and Wilding, 2008, Yick and Wilding, 2014).

By this view, the findings in this experiment provide what is, to the best of our knowledge, the first evidence in the same data set for processes operating at both stages: there were reliable differences between the scalp distributions of the imagine and perceive target old/new effects in time windows preceding and following the 500–800 msec window in which left-parietal ERP old/new effects were evident in both conditions. While the early divergences between the scalp distributions of the perceive and imagine ERP old/new effects comprise strong evidence in support of neural reinstatement accounts, these data do not allow confident claims about what is being reinstated. In the two target designations there were differences at the time of encoding in the perceptual content to which participants were exposed, as well as, presumably, in the cognitive operations that were engaged. The context-sensitivity at the time of retrieval might reflect one or other of these elements, or some combination of the two.

It is possible, however, to consider accounts motivated by previous work and apply them to some of the data reported here. In the 500–800 msec time window the scalp distribution of the imagine old/new effect bears similarities with the temporally similar frontal effect reported by Johnson et al. (2008) for words encoded in sentences. A potentially complementary set of findings comes from functional Magnetic Resonance Imagining (fMRI) studies, where increased anterior prefrontal cortex (PFC) activation has been associated with the recovery of self-generated, compared to externally presented information (Simons et al., 2005, Turner et al., 2008). If the anterior projections in the ERP studies described above reflect activity in PFC, the presence of the effect for the sentence generation condition reported by Johnson and colleagues and the imagine condition in this experiment is accommodated easily under a self-generated account.

However, for the 500–800 msec epoch this interpretation does not, at least at a first pass, sit comfortably with the fact that these anterior distributions are reminiscent of those reported previously for faces and objects (Galli and Otten, 2010, MacKenzie and Donaldson, 2007). Delineating the functional significance of content-specific old/new effects requires further investigations in which tighter control over the differences between encoding conditions is exercised than has been achieved to date.

It is also noteworthy that the effects in the study here were obtained in tasks where discrimination between contexts was required. This was not the case in the study by Johnson et al. where Remember and Know judgments were required (Johnson & Rugg, 2007). This is important because of the possibility that the content-specific signatures observed in this study are a consequence of the specific binary context discrimination participants made. It follows from this supposition that the neural signature associated with a particular content might vary according to the specific discrimination that is required. This observation is at one level a reiteration of a key assumption of the source monitoring framework, according to which a strategic assessment of memory characteristics is guided by which characteristics are diagnostic for the particular discrimination that needs to be made (Johnson et al., 1993, Johnson et al., 1981). Wilding (1999) noted that this framework could include what information is recovered from memory depending on task demands, as well as the ways in which recovered content is assessed, thereby offering a means of interpreting both early (300–500) and late (800–1100) divergences in this experiment. For the latter, the divergences observed here might reflect different assessment processes, or the same kinds of assessment operating over different representations (Rugg, 1994).

Finally, the source monitoring framework is also relevant to a consideration of the findings reported by Johansson, Stenberg, Lindberg, and Rosen (2002). Using very similar encoding tasks to those employed in this study, they observed no differences between the scalp distributions of ERP old/new effects. In their experiments participants made forced-choice memory judgments and - critically - response accuracy approached ceiling. This high level of accuracy is important because it offers a plausible explanation for the apparent disconnect between their findings and those reported here in a somewhat similar paradigm. Presumably the incentive to prioritise recovery of certain kinds of contents according to the discrimination that is required diminishes if diagnostic information is readily available.

In conclusion, these data extend the range of circumstances under which ERPs index retrieval in a content-sensitive manner. While indices occurring in parallel or after an effect that has been linked closely with recollection might reflect monitoring operations that are either content- or task-specific, earlier divergences (before 500 msec post-stimulus) are candidates for reinstatement of content-specific encoding operations. Evidence for processes operating at these two stages has not, to our knowledge, been reported before in the same study. In addition, these outcomes broaden the scope for ERPs to be employed to investigate questions about (among other factors) retrieval control, retrieval suppression and content-specific retrieval impairments.

## Figures and Tables

**Table 1:** The probabilities of correct responses (PCorr) to Targets, Non-Targets and New test words in the Imagine and the Perceive target designations. Also shown are reaction times (RT) in milliseconds for these response categories. Standard deviations are in parentheses.

Item type	Imagine		Perceive	
	PCorr	RT	PCorr	RT
Target	.76 (.12)	1111 (197)	.73 (.15)	1044 (170)
Non-target	.87 (.08)	1101 (230)	.86 (.07)	1090 (176)
New	.92 (.08)	1024 (189)	.94 (.07)	1009 (206)

## Footnotes

1 Assessments of content-sensitivity were restricted to the ERP old/new effects associated with correct responses to targets. Correct responses to non-targets can be made when participants have little or no memory for these items, because they are directed to identify non-targets and new test items via the same response in exclusion task test phases. This outcome will result in a level of contamination in the averaged ERPs associated with correct response to non-targets that will be diminished markedly for correct responses to targets.

2 For discussions of this method, see (McCarthy and Wood, 1985, Urbach and Kutas, 2002, Wilding, 2006).

3 The extended time-course and sustained distribution of the divergences is consistent with the findings in previous studies (Bridger et al., 2009, Rosburg et al., 2011, Wilding, 1999).
